# Factors associated with physical activity engagement among adolescents in a southeastern region of Spain

**DOI:** 10.1016/j.pmedr.2025.103063

**Published:** 2025-04-06

**Authors:** José Ramón Solano Lorente, Mario Diaz Cutillas, Sara Luna Rivas, Francisco José Rivera de los Santos, Aníbal Nieto Diaz, Olga Monteagudo Piqueras

**Affiliations:** a University of Murcia, Spain; b University of Seville, Spain

**Keywords:** Physical activity, Adolescents, Sedentary lifestyle, Childhood obesity, Quality of life, Socioeconomic factors

## Abstract

**Objective:**

To identify factors associated with physical activity engagement among adolescents in southeastern Spain.

**Methods:**

A cross-sectional study was conducted with a representative sample of 1817 adolescents from the Region of Murcia, using data from the 2018 HBSC Spain study. Two logistic regression models were analyzed. Model 1 included the full sample, with the dependent variable being compliance with the World Health Organization's physical activity recommendations (WHO-PAR), which include both moderate and vigorous physical activity. Model 2 focused on adolescents who did not meet the WHO-PAR but reported engaging in vigorous physical activity during leisure time at least four times per week.

**Results:**

In total, 19.7 % of adolescents met the WHO-PAR. Among those who did not, 24.8 % engaged frequently in vigorous physical activity (≥4 times per week). In Model 1, being female (OR 0.43), older age (OR 0.87), and overweight/obesity (OR 0.59) were associated with lower odds of meeting the WHO-PAR. High family affluence (OR 1.71) and high perceived quality of life (OR 1.99) were associated with greater odds. In Model 2, being male (OR 1.94), high family affluence (OR 1.55), and high quality of life (OR 1.98) increased the likelihood of engaging in vigorous physical activity, while overweight/obesity (OR 0.61) decreased it.

**Conclusions:**

Not meeting the WHO physical activity recommendations and not engaging in vigorous activity were associated with low family affluence, being female, overweight/obesity, and low or medium perceived quality of life. These factors should be addressed in interventions to promote adolescent physical activity.

## Highlights

• Only **19.7 % of adolescents** in the Region of Murcia met WHO physical activity recommendations.
• Just **33.7 % engaged in vigorous physical activity** at least four times per week.
• **Girls and adolescents with overweight or obesity** were less likely to meet activity guidelines.
• **Higher health-related quality of life and greater family affluence** were associated with increased physical activity.
• **Targeted interventions** are needed to address gender disparities and socioeconomic barriers.

## 1 Introduction

Physical activity, defined as any intentional bodily movement that increases energy expenditure, is essential for interacting with the environment (Guillén and Linares, 2002). It includes sports and recreational activities but also daily tasks, and it is classified by purpose and intensity (González-Jurado, 2004). Moderate activity, such as brisk walking or dancing, increases breathing and heart rate, while vigorous activity, such as running or skating, causes heavy breathing, sweating, and fatigue (Airasca and Giardini, 2009). Its benefits span physical, psychological, and social domains and are critical for the prevention and treatment of various health conditions. Among adolescents, it improves cardiovascular, respiratory, digestive, and endocrine systems, strengthens the musculoskeletal system, enhances flexibility, and reduces cholesterol, triglycerides, obesity, and body fat. Psychologically, it increases self-confidence, well-being, and cognitive function, positively impacting mental health, stress tolerance, self-concept, and self-esteem (Ramírez et al., 2004). It also offers protection against personality disorders, social anxiety, and deficits in social skills (Penedo and Dahn, 2005). Furthermore, the release of endorphins and inhibition of nerve fibers during physical activity produce analgesia, a sense of well-being, and relaxation, thereby improving adolescents' quality of life (Pérez-Samaniego, 2003).

Despite these many benefits, physical activity levels remain insufficient due to sedentary work habits and fast-paced lifestyles that reduce both free time and motivation (Owen et al., 2000). According to Spain's 2022 Living Conditions Survey (INE) (Estadística, 2024), only 37.7 % of individuals over 16 engage in regular physical activity during their free time. The Region of Murcia shows the lowest rate among Spanish regions, at 28.6 %, well below the 34.2 % in the Valencian Community. In youth, the ALADINO and PASOS 2024 studies (Gasol Foundation, 2024) indicate that about 67.4 % of boys and 58.6 % of girls meet physical activity recommendations, while 23.8 % of boys and 24.7 % of girls are sedentary. The gender gap in physical activity widens during preadolescence and adolescence, highlighting the need to address the specific barriers girls face. To this end, Spain's “National Strategic Plan for the Reduction of Childhood Obesity (2022–2030)” (Comisionado para la lucha contra la pobreza infantil, 2023) proposes increasing physical education time in schools, integrating physical activity into the curriculum, and improving access to safe spaces for its practice. This plan draws on the ecological model of health behaviour, which considers the interaction of individual, social, and environmental factors as determinants of adolescents' physical activity.

The Health Behaviour in School-aged Children (HBSC) study is an international research initiative launched in 1982 to examine health-related behaviors in school-aged adolescents. Supported by the 10.13039/100004423WHO, it collects data every four years in multiple countries to track trends and compare adolescent lifestyles. This framework has generated numerous studies exploring the interrelationship between physical activity, body image, and various socioeconomic and health factors in adolescents. For instance, Ramos et al. (2019) analyzed how body image satisfaction and perceived overweight influence adolescent mental health. Moreno-Maldonado et al. (2019) examined how both objective and subjective socioeconomic factors directly impact adolescent health and lifestyles, emphasizing perceived affluence as a key mediator between socioeconomic status and health. In this context, Ramos et al. (2016) highlighted the need to promote active lifestyles among adolescents, especially those from lower socioeconomic backgrounds.

Although previous studies have addressed this issue (European Health Equity Dataset [Internet], 2024), it is necessary to update and contextualize findings in specific regions with particularly low physical activity rates, such as the Region of Murcia, as reported by recent INE data. Moreover, few studies simultaneously examine both compliance with WHO recommendations and participation in vigorous leisure-time physical activity. Therefore, it is essential to understand the factors that influence adolescents' behaviour—identifying which are modifiable and which are not—and analyze their implications for health. It is important to note that this study does not include muscle-strengthening exercise, as the HBSC questionnaire does not contain specific items on this dimension of physical activity.

## 2 Methods

### 2.1 Study design and population

This is a cross-sectional study based on data from the 2018 HBSC survey (pre-pandemic period) conducted in the Region of Murcia. The sample consisted of 1817 adolescents aged 11 to 18 years, representative of the regional population.

### 2.2 Study variables

*Dependent variable 1*: Compliance with WHO recommendations (Yes/No). This variable derives from the 2018 HBSC questionnaire item that asks adolescents how often they engage in at least 60 min of moderate-to-vigorous physical activity (MVPA) per day. Response options range from “0 days” to “7 days.” The variable was dichotomized as “Yes” for those reporting 60 min of MVPA on all 7 days of the week, in line with WHO recommendations, and “No” for those who did not reach that level (fewer than 7 days).

*Dependent variable 2*: Vigorous leisure-time physical activity. This variable is also based on the 2018 HBSC questionnaire, which asks adolescents how often they engage in physical activity during their free time that makes them sweat or breathe hard. Response categories include “Never,” “Less than once a week,” “Once a week,” “2–3 times a week,” “4–6 times a week,” and “Every day.” The variable was dichotomized as “Yes” for those who reported engaging in vigorous activity 4–6 times a week or more, and “No” for those with lower frequency.

*Explanatory variables*: sex; age (in years, treated as a continuous variable); family affluence, measured by the Family Affluence Scale (FAS), based on ownership of household items such as bedrooms or computers (Currie et al., 2008) and categorized as low, medium, or high; body mass index (BMI), calculated from self-reported height and weight (weight/height^2^) and categorized using the cut-off points proposed by Cole et al. (2000) and Cole et al. (2007) to define underweight/normal weight and overweight/obesity; and health-related quality of life, assessed using the KIDSCREEN instrument validated in Spanish (Ravens-Sieberer and European kidscreen group, 2006). The variables were selected based on existing literature on determinants of physical activity in adolescents and their availability in the HBSC questionnaire. It is important to note that the HBSC does not include specific items on muscle-strengthening exercises; therefore, this dimension was not considered in the present analysis.

### 2.3 Statistical models and analysis

Physical activity engagement was explored using two logistic regression models. Model 1 included the full sample and examined compliance with WHO physical activity recommendations. Model 2 included only those adolescents who did not meet the WHO recommendations, analyzing their engagement in vigorous leisure-time physical activity. The explanatory variables for both models were: sex, age, family affluence, body mass index, and health-related quality of life (KIDSCREEN-10). A preliminary bivariate analysis was conducted to assess associations between dependent and explanatory variables in both models.

Data analysis was performed using R statistical software. Each logistic regression model included sensitivity (proportion of correctly identified positive cases) and specificity (proportion of correctly identified negative cases), based on the optimal point of the ROC curve, which simultaneously maximizes both sensitivity and specificity. In addition, the area under the curve (AUC) was used to assess discriminative ability, and the Akaike Information Criterion (AIC) was reported to evaluate model fit and complexity. Multicollinearity was assessed using variance inflation factor (VIF) analysis, and all values were considered acceptable (<2 in all cases).

## 3 Results

The total sample analyzed included 1817 adolescents, whose characteristics are detailed in Table 1. Of the total, 49.3 % were girls, with ages ranging from 11 to 18 years. Regarding family affluence, 53.4 % were classified in the medium level. In terms of body mass index (BMI), 19.0 % of adolescents were overweight or obese. With respect to physical activity, 19.7 % met the WHO's daily recommendation of 60 min of physical activity (1446 adolescents). As for the frequency of vigorous leisure-time physical activity, 33.7 % reported engaging in it 4 to 6 times per week or more (613 adolescents). Finally, 32.6 % of the surveyed adolescents reported a high level of perceived health-related quality of life (592 adolescents). Although odds ratios (ORs) are presented as the main measure of association, we acknowledge that, given the observed prevalence rates, the use of adjusted prevalence ratios (APRs) could be a methodologically more appropriate alternative. This possibility will be considered in future analyses.

**Table 1:** Sociodemographic and Health-Related Characteristics of Adolescents (Aged 11–18) in the Region of Murcia, Health Behaviour in School-aged Children (HBSC) Study, 2018.

Variable	Categories	N	%
Gender	Boys	922	50.7
	Girls	895	49.3
Age Group	11–12 years	455	25.0
	13–14 years	544	29.9
	15–16 years	474	26.1
	17–18 years	344	18.9
Purchasing Power	Low	304	16.7
	Medium	764	42.0
	High	364	20.0
	Not Available (NA)	385	21.2
BMI Classification	Underweight/Normal weight	1266	69.7
	Overweight/Obesity	346	19.0
	Not Available (NA)	205	11.3
WHO Recommendations	Below WHO recommendation	1446	79.6
	Daily	358	19.7
	Not Available (NA)	13	0.7
Vigorous Activity in Leisure Time	Up to 2–3 times a week	1119	61.6
	4–6 times a week or more	613	33.7
	Not Available (NA)	85	4.7
Quality of Life	Low/Medium	906	49.9
	High	592	32.6
	Not Available (NA)	319	17.6

### 3.1 Results of model 1 for the total adolescent sample

From the total sample, 358 adolescents (19.8 %) met the WHO physical activity recommendations. Table 2 presents the bivariate analysis of factors associated with adherence to these recommendations. The results indicate that being male, being older, belonging to families with high affluence, and reporting a high quality of life were significantly associated with meeting the physical activity recommendations. Conversely, adolescents with overweight or obesity showed a lower likelihood of meeting these recommendations.

**Table 2:** Bivariate Associations Between Sociodemographic and Health Factors and Compliance with WHO Physical Activity Recommendations Among Adolescents.

Variable	Categories	N	% in Category	*p*-value (Chi-s)
Gender	Boys	233	25.3	<0.001
	Girls	125	14.0	
Age Group	11–12 years	123	27.0	<0.001
	13–14 years	116	21.3	
	15–16 years	85	17.9	
	17–18 years	34	9.9	
Purchasing Power	Low	44	14.5	0.004
	Medium	143	18.7	
	High	90	24.7	
BMI Classification	Underweight/Normal weight	259	20.5	0.152
	Overweight/Obesity	59	17.1	
Vigorous Activity in Leisure Time	Up to 2–3 times a week	88	7.9	<0.001
	4–6 times a week or more	250	40.8	
Quality of Life	Low/Medium	133	14.7	<0.001
	High	179	30.2	

**Fig. 1:**
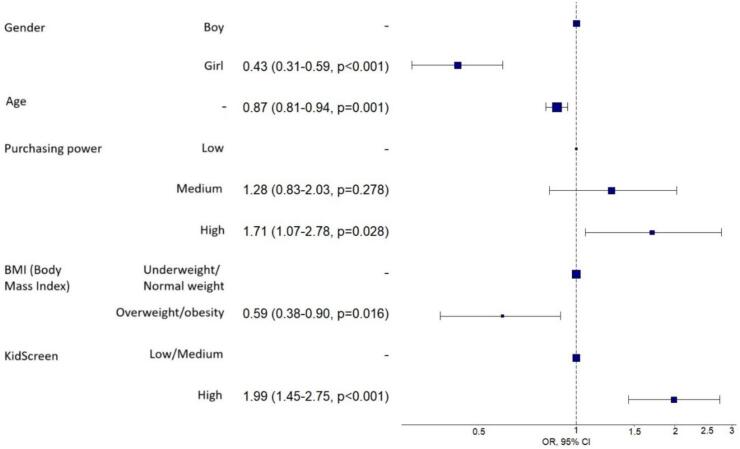
Odds Ratios and 95 % Confidence Intervals for Factors Associated with Meeting WHO Physical Activity Recommendations Among Adolescents in the Region of Murcia, Health Behaviour in School-aged Children (HBSC) Study, 2018.

The multivariate analysis, whose results are presented in Fig. 1, shows that being male (OR 0.43; 95 % CI: 0.31–0.59; *p* < 0.001) and being older (OR 0.87; 95 % CI: 0.81–0.94; *p* = 0.001) significantly decrease the likelihood of meeting the recommendations. Having overweight or obesity (OR 0.59; 95 % CI: 0.38–0.90; *p* = 0.016) also reduces the probability of compliance. On the other hand, reporting a high quality of life significantly increases the likelihood of meeting the recommendations (OR 1.99; 95 % CI: 1.45–2.75; *p* < 0.001). The model showed a specificity of 63.1 %, sensitivity of 68.8 %, an AUC of 70.1 %, and an AIC of 1044.9, indicating acceptable fit and discriminative capacity. AUC and AIC are commonly used as validation metrics for model performance in studies of this kind.

### 3.2 Results of model 2 for adolescents not meeting who physical activity recommendations

Among the 1446 adolescents who did not meet the WHO recommendations, 24.9 % (360 adolescents) reported engaging in vigorous physical activity 4 to 6 times per week or more. Table 3 describes the characteristics of this subsample. The bivariate analyses, presented in Table 4, suggest that being male, being aged 15 to 18 years, and reporting a high quality of life are significantly associated with a higher likelihood of engaging in vigorous physical activity. No significant differences were observed in family affluence.

**Table 3:** Characteristics of Adolescents Not Meeting WHO Physical Activity Recommendations in the Region of Murcia, Health Behaviour in School-aged Children (HBSC) Study, 2018.

Variable	Categories	N	%
Gender	Boys	685	47.4
	Girls	761	52.6
Age Group	11–12 years	330	22.8
	13–14 years	422	29.2
	15–16 years	386	26.7
	17–18 years	308	21.3
Purchasing Power	Low	256	17.7
	Medium	617	42.7
	High	274	18.9
	Not Available (NA)	299	20.7
BMI Classification	Underweight/Normal weight	1000	69.2
	Overweight/Obesity	286	19.8
	Not Available (NA)	160	11.1
Vigorous Activity in Leisure Time	Up to 2–3 times a week	1026	71.0
	4–6 times a week or more	360	24.9
	Not Available (NA)	60	4.1
Quality of Life	Low/Medium	769	53.2
	High	410	28.4
	Not Available (NA)	267	18.5

**Table 4:** Bivariate Analysis of Factors Associated with Vigorous Physical Activity Among Adolescents Not Meeting WHO Recommendations.

Variable	Categories	N	% in Category	p-value (Chi-s)
Gender	Boys	230	33.6	<0.001
	Girls	130	17.1	
Age Group	11–12 years	93	28.2	0.010
	13–14 years	118	28.0	
	15–16 years	91	23.6	
	17–18 years	58	18.8	
Purchasing Power	Low	52	20.3	0.012
	Medium	158	25.6	
	High	86	31.4	
BMI Classification	Underweight/Normal weight	262	26.2	0.022
	Overweight/Obesity	57	19.9	
Quality of Life	Low/Medium	164	21.3	<0.001
	High	137	33.4	

In Table 4, the bivariate analysis of adolescents who do not meet WHO recommendations but engage in vigorous physical activity during their leisure time is presented.

**Fig. 2:**
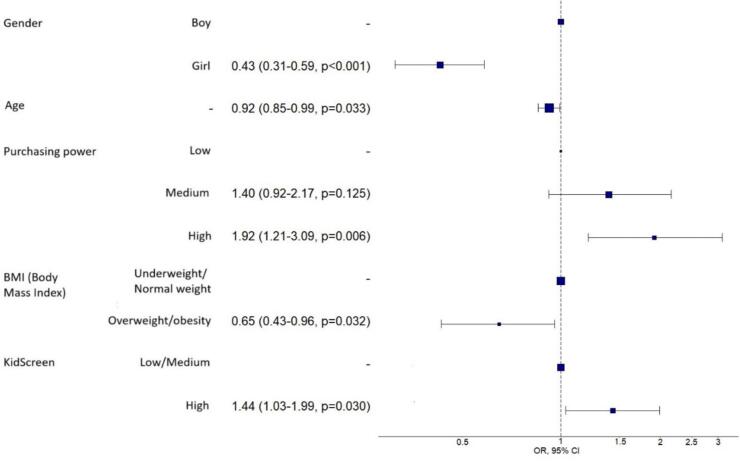
Odds Ratios and 95 % Confidence Intervals for Factors Associated with Vigorous Leisure-Time Physical Activity Among Adolescents Not Meeting WHO Recommendations, Health Behaviour in School-aged Children (HBSC) Study, 2018.

The multivariate analysis, illustrated in Fig. 2, indicates that being male (OR 0.43; 95 % CI: 0.31–0.59; *p* < 0.001) and being older (OR 0.92; 95 % CI: 0.85–0.99; *p* = 0.033) significantly increase the likelihood of engaging in vigorous physical activity during leisure time. Having overweight or obesity reduces this likelihood (OR 0.45; 95 % CI: 0.30–0.69; *p* = 0.032), while reporting a high quality of life is significantly associated with an increase in vigorous physical activity (OR 1.44; 95 % CI: 1.03–1.99; *p* = 0.030). The model showed a specificity of 63.7 %, sensitivity of 64.7 %, an AUC of 66.9 %, and an AIC of 993.3, indicating acceptable fit and discriminative capacity. AUC and AIC are commonly used as validation metrics for model performance in studies of this kind.

## 4 Discussion

This analysis, framed within the HBSC-Spain project, aimed to identify factors associated with compliance with WHO physical activity recommendations and engagement in vigorous physical activity among adolescents in the Region of Murcia. The findings reflect complex, interrelated patterns that must be interpreted through a socio-ecological lens, as physical activity behaviour is influenced by individual, social, and environmental factors.

From an individual perspective, our results are consistent with previous studies showing that girls are less likely than boys to meet physical activity recommendations and engage in vigorous activity. The HBSC report (HBSC, 2022) highlights specific barriers faced by adolescent girls, including perceived lack of competence, social pressure, and limited free time due to household or academic responsibilities. Adolescents with overweight or obesity also had lower odds of meeting the recommendations, likely influenced by a cycle of inactivity and psychosocial barriers such as body image concerns or fear of social judgment (Larson et al., 2022). These findings underscore the importance of interventions that promote self-esteem, reduce stigma, and create supportive social environments.

At the social level, high perceived quality of life and greater family affluence were positively associated with physical activity engagement. This aligns with previous research suggesting that material resources and emotional support from families contribute to increased participation (Silva et al., 2019). Enjoyment also plays a key role: Hughes et al. (Hughes et al., 2022) and Tammaine et al. (Tammaine et al., 2005) noted that adolescents who find physical activity enjoyable are more likely to maintain regular habits. Interventions that foster early positive experiences with physical activity can therefore have long-term effects.

At the community level, our findings reinforce the relevance of structural factors. In line with the ecological model of health behaviour, accessibility to safe, affordable, and youth-friendly spaces can determine adolescents' capacity to engage in regular activity. Martínez-Moyá et al. (Martínez-Moyá et al., 2014) showed that reducing economic barriers and improving infrastructure significantly increases participation. Ruiz-Trasserra et al. (Ruiz-Trasserra et al., 2017) emphasized the need for policies that address inequalities and promote equitable access to physical activity opportunities. Furthermore, Burchfiel et al. (Burchfiel et al., 2013) reported that adolescents engaging in moderate-to-vigorous physical activity are 10 % less likely to be obese. Even modest increases in weekly activity can lead to meaningful reductions in BMI, reinforcing the cumulative benefits of regular exercise.

The use of data from the 2018 HBSC study, a pre-pandemic period, provided a stable baseline unaffected by behavioral disruptions due to COVID-19. However, research such as Stockwell et al. (Stockwell et al., 2021) has shown that the pandemic significantly reduced physical activity levels, especially among adolescents. Future analyses using HBSC, 2022 data will be essential to assess the long-term effects of the pandemic.

## 5 Limitations

This study has several limitations. First, the cross-sectional design does not allow for causal inferences between the variables analyzed. Second, all data are based on self-reports, which may introduce biases such as overestimation or underestimation of physical activity and other key measures. Third, the sample only includes school-attending adolescents from the Region of Murcia, which limits the generalizability of the findings to other populations or regions. In addition, the HBSC questionnaire does not include items on muscle-strengthening activities, and this component of physical activity was therefore not addressed in our analysis. Lastly, although excluding pandemic data ensures a more stable behavioral baseline, it also restricts the temporal relevance of the findings.

### 5.1 Strengths and practical implications

Among the main strengths of this study are the use of a large, representative sample and the application of robust statistical models. The results contribute to a better understanding of the social and health-related determinants of physical activity among adolescents and offer valuable guidance for public health strategies. Interventions should prioritize reducing perceived and structural barriers among girls, adolescents with overweight or obesity, and those from less affluent families. Policies should aim to improve access to physical activity in all settings—school, community, and home—and promote early enjoyment of physical activity, which has been shown to predict long-term adherence. The ecological model of health behaviour should guide intervention design, incorporating individual, social, and environmental components to maximize impact.

### 5.2 Future Research

Future studies should employ longitudinal designs to explore causal relationships between the variables examined. It will also be essential to evaluate the effectiveness of multi-level interventions that target adolescents from different socioeconomic and demographic backgrounds. Finally, new analyses based on the 2022 HBSC data should assess the long-term impact of the COVID-19 pandemic on adolescents' physical activity behaviors and their broader implications for physical and mental health.

## 6 Conclusions

This study identified individual, social, and community-level factors associated with adherence to physical activity recommendations and engagement in vigorous physical activity among adolescents in the Region of Murcia. The findings reveal clear gender disparities, the negative influence of overweight and obesity, and the protective role of social support and perceived quality of life in fostering an active lifestyle. These results underscore the importance of developing multifaceted interventions that integrate individual, social, and structural components, with a particular focus on vulnerable groups such as adolescent girls, those with lower socioeconomic status, and those with excess weight.

The implications for preventive medicine are substantial, highlighting the urgent need for inclusive public policies and school-based strategies that guarantee equitable access to physical activity opportunities. This study also contributes to a deeper understanding of the determinants of adolescent physical activity and provides a valuable framework for future longitudinal research to assess the effectiveness of socio-ecological interventions. Finally, it is essential to consider the potential impact of recent societal disruptions—such as the COVID-19 pandemic—on adolescents' physical activity habits and overall health.

## Data collection

The data were collected as part of the 2018 HBSC survey in Spain, specifically in the Region of Murcia, between March and May 2018.

## Transparency declaration

The corresponding author, on behalf of all co-signing authors, ensures the accuracy, transparency, and honesty of the data and information contained in the study; that no relevant information has been omitted; and that all discrepancies among authors have been appropriately resolved and described.

## CRediT authorship contribution statement

**José Ramón Solano Lorente:** Writing – review & editing, Writing – original draft, Formal analysis, Conceptualization. **Mario Diaz Cutillas:** Software, Formal analysis, Data curation. **Sara Luna Rivas:** Supervision, Resources, Project administration. **Francisco José Rivera de los Santos:** Investigation, Funding acquisition. **Aníbal Nieto Diaz:** Visualization, Validation. **Olga Monteagudo Piqueras:** Supervision, Formal analysis.

## Funding

This work was supported by the National Plan on Drugs (Government of Spain).

## Declaration of competing interest

The authors declare the following financial interests/personal relationships which may be considered as potential competing interests: Jose Ramon Solano Lorente reports writing assistance was provided by University of Murcia. Olga Monteagudo Piqueras reports a relationship with University of Murcia that includes: non-financial support. Olga Monteagudo Piqueras has patent licensed to Estudio HBSC (Health Behaviour in School-aged Children). If there are other authors, they declare that they have no known competing financial interests or personal relationships that could have appeared to influence the work reported in this paper.

## Data Availability

Data will be made available on request.

## Appendix A Supplementary data

Supplementary Table 1. Summary of existing evidence, study contributions, and implications for adolescent physical activity promotion in line with WHO guidelines. Image 1
