# Supplementary material for: Factors associated with physical activity engagement among adolescents in a southeastern region of Spain
Source: Prev Med Rep. 2025 Apr 6;53:103063. doi: 10.1016/j.pmedr.2025.103063 (PMC12008529; doi:10.1016/j.pmedr.2025.103063)
Supplement: Supplementary file 1 — Supplementary Table 1. Summary of existing evidence, study contributions, and implications for adolescent physical activity promotion in line with WHO guidelines. [file mmc1.docx]

EXPLANATORY SUMMARY

| **What is known about the topic?** | Most adolescents do not meet the WHO minimum daily physical activity recommendations. This negatively impacts their physical and mental health, increasing chronic disease risks and lowering their quality of life. |
| --- | --- |
| **What does this study add to the literature?** | It identifies specific demographic and socioeconomic factors influencing adherence to physical activity recommendations and their relationship with quality of life. |
| **What are the implications of the results?** | Policies should promote physical activity among adolescents, particularly those with overweight or obesity and from lower socioeconomic groups, to improve their quality of life and overall well-being. |
